# Patient satisfaction with calcipotriol/betamethasone dipropionate cutaneous foam for the treatment of plaque psoriasis: The LION real‐life multicenter prospective observational cohort study

**DOI:** 10.1111/dth.15077

**Published:** 2021-08-09

**Authors:** Anna Campanati, Laura Atzori, Concetta Potenza, Giovanni Damiani, Luca Bianchi, Monica Corazza, Rossana Tiberio, Francesca Prignano, Giuseppe Argenziano, Maria Concetta Fargnoli, Luca Stingeni, Annamaria Mazzotta, Ornella De Pità, Carlo Mazzatenta, Claudio Feliciani, Massimo Donini, Annamaria Offidani, Ketty Peris, LION study group, Franco Rongioletti, Paolo Pigatto, Giovanni Cruciani, Clara De Simone, Giacomo Caldarola, Marta Grimaldi, Jasmine Anedda, Nicoletta Bernardini, Anna Marchesiello, Chiara Franchi, Annunziata Dattola, Elisabetta Botti, Pierantonia Zedde, Edoardo Cammarata, Giulia Radi, Elia Rosi, Alina De Rosa, Maria Esposito, Rossella Marietti, Francesca Lupi, Marta Grazzini, Di Maria Domenico, Marco Brusasco, Fabiana Gai

**Affiliations:** ^1^ Dermatology Unit, Department of Clinical and Molecular Sciences Polytechnic Marche University Ancona Italy; ^2^ Department of Medical Sciences and Public Health, Dermatology Clinic University of Cagliari Cagliari Italy; ^3^ Dermatology Unit Daniele Innocenzi, Department of Medical‐Surgical Sciences and Biotechnologies Sapienza University of Rome, Fiorini Hospital, Polo Pontino Terracina Italy; ^4^ Clinical Dermatology Istituto Ortopedico Galeazzi IRCCS Milan Italy; ^5^ Department of Dermatology University of Rome Tor Vergata Rome Italy; ^6^ Section of Dermatology, Department of Medical Sciences University of Ferrara Ferrara Italy; ^7^ Dermatologic Clinic, Department of Health Science University of Eastern Piedmont Novara Italy; ^8^ Unit of Dermatology, Department of Health Science University of Florence Florence Italy; ^9^ Dermatology Unit University of Campania Luigi Vanvitelli Naples Italy; ^10^ Dermatology, Department of Biotechnological and Applied Clinical Sciences University of L'Aquila L'Aquila Italy; ^11^ Dermatology Section, Department of Medicine University of Perugia Perugia Italy; ^12^ Dermatology Unit San Camillo‐Forlanini Hospital Rome Italy; ^13^ Clinical Pathology and Immune Inflammatory Disease of the Skin Cristo Re Hospital Rome Italy; ^14^ Dermatology Unit Lucca Azienda USL Toscana Nord Ovest Pisa Italy; ^15^ Section of Dermatology, Department of Clinical and Experimental Medicine University of Parma Parma Italy; ^16^ Department of Dermatology SS Giovanni E Paolo Civil Hospital Venice Italy; ^17^ UOC di Dermatologia Fondazione Policlinico Universitario A. Gemelli IRCCS Rome Italy

**Keywords:** psoriasis, therapy‐topical

## Abstract

Topical treatment is the mainstay for mild or moderate psoriasis, but patients are generally little satisfied. Calcipotriol/betamethasone dipropionate (Cal/BD) cutaneous foam has shown to improve signs and symptoms in plaque psoriasis patients. This study assessed patient's satisfaction with Cal/BD foam in a real‐life Italian dermatological clinical practice. A multicenter, 4‐week observational prospective cohort study enrolled, in 17 Italian dermatology clinics, adult patients with plaque psoriasis on the body and/or scalp. Treatment satisfaction was assessed by 9‐item Treatment Satisfaction Questionnaire for Medication (TSQM‐9), preference over previous treatments by Patient Preference Questionnaire (PPQ), and change in disease state by Psoriasis Area Severity Index (PASI). Overall 256 patients were eligible, with a mean (SD) age of 55.6 (15.4) years, 59.4% were males. Psoriasis severity was mild in 52.0% of patients, moderate in 43.3%, and severe in 4.7%. Scalp involvement was present in 36.7% of patients. Previous antipsoriatic treatments had been received by 80.5% of patients. TSQM‐9 median (25th–75th percentile) scores were 83.3 (66.7–88.9) for effectiveness, 77.8 (66.7–88.9) for convenience, and 78.6 (64.3–92.9) for global satisfaction. Mean (SD) PASI value decreased from 7.3 (4.8) to 2.1 (2.7) after 4 weeks. More than 90% of patients previously treated for psoriasis evaluated the Cal/BD foam more effective, easier to use and better tolerated compared to previous topical treatments at PPQ. This observational study provides real‐life evidence of a high level of satisfaction with effectiveness and convenience of the Cal/BD foam in a cohort of plaque psoriasis patients, with an objective improvement in PASI.

## 1 INTRODUCTION

Psoriasis is a common chronic inflammatory multisystem disease with a strong genetic predisposition and an immune pathogenetic component.^1^, ^2^, ^3^ Approximately 90% of patients affected with psoriasis have plaque psoriasis.^4^ Psoriasis is recognized to severely impact patients' quality of life (QoL) largely due the appearance of skin lesions and the troublesome itch.^5^, ^6^

Topical treatment is the mainstay for mild or moderate psoriasis, also in addition to phototherapy, systemic conventional or biologic therapy.^7^ In general, psoriasis patients are not very satisfied with their treatment and significantly least those receiving topical treatment, especially when affected with moderate disease.^8^, ^9^

A topical once‐daily, fixed‐dose combination (FDC) of 50 μg/g of calcipotriol (Cal)—a synthetic vitamin D3 analog—with 0.5 mg/g of the synthetic corticosteroid betamethasone dipropionate (BD) in a cutaneous foam formulation (LEO Pharma) has been introduced in the treatment armamentarium for plaque psoriasis in adults. In phase II–III studies, Cal/BD foam obtained significantly greater improvements in Psoriasis Area Severity Index (PASI), Dermatology Life‐Quality Index (DLQI), and total clinical scores than placebo, BD topical comparators, or a gel and ointment Cal/BD formulation, with a favorable safety profile.^10^, ^11^, ^12^, ^13^ In the foam aerosol, the active ingredients are dissolved in volatile propellants that evaporate when the product is applied to the skin, creating a foam layer containing a supersaturation of the actives that enables a greater penetration of the drug through the epidermis. This is believed to account for the higher efficacy shown by the cutaneous foam compared to the gel and ointment Cal/BD formulation.^12^

Confirmation of a drug effectiveness in the real‐life setting is becoming increasingly relevant.^14^ Factors such as patient preference and satisfaction with the prescribed therapy—which have a major impact on adherence to treatment—are paramount in how treatments perform in everyday clinical practice. Measurement of these parameters is becoming increasingly valuable because it can provide data on how satisfaction improves adherence and influences treatment choice.

The LION study was aimed at providing real‐life evidence of the satisfaction of plaque psoriasis patients with Cal/BD cutaneous foam treatment in the Italian dermatological clinical practice.

## 2 PATIENTS AND METHODS

The LION study was an Italian multicenter observational prospective cohort study conducted in 17 dermatology clinics across different Italian regions. The observational period was framed between the enrolment visit, performed prior to the initiation of Cal/BD foam treatment, and the follow‐up visit, performed as per clinical practice 4 ± 1 weeks after treatment initiation or at patient's exit from study, whichever occurred first. Data sources for primary data collection included medical records and patient‐reported outcome (PRO) questionnaires.

### 2.1 Patients

Patients aged ≥18 years with plaque psoriasis on the body and/or scalp, providing written informed consent before the data collection and assigned to treatment with Cal/BD foam as per ordinary clinical practice were enrolled in the study. Cal/BD foam was prescribed according to the summary of product characteristics (SmPC) clinical information according to the observational nature of the study. Patients did not undergo any additional diagnostic or monitoring procedures. Patients treated with systemic conventional or biologic agents within 16 weeks before enrolment, or under treatment with other therapies for psoriasis in an interventional clinical trial were excluded, as were pregnant or breast‐feeding women and patients unable to understand and fill in the study questionnaires.

The use of concurrent phototherapy or topical corticosteroid did not represent an exclusion criterion.

### 2.2 Study objectives

The primary objective of the LION study was to describe the patients' satisfaction with Cal/BD cutaneous foam treatment after 4 weeks of therapy in the clinical practice setting, by means of the 9‐item Treatment Satisfaction Questionnaire for Medication (TSQM‐9).^15^, ^16^

Secondary objectives of the study were to describe (I) factors potentially associated to the satisfaction with Cal/BD foam treatment, such as age, gender, and duration and severity of psoriasis, (II) patients' treatment preference using the Patient Preference Questionnaire (PPQ),^17^ and (III) the change in the PASI score^18^ following treatment.

### 2.3 Sample size

According to feasibility considerations, assuming the inclusion of 250 psoriatic patients (20% of whom possibly not evaluable for the primary analysis due to non‐available data, dropout, or failure in meeting the eligibility criteria), we estimated the achievable precision of the primary endpoint on 200 patients.

### 2.4 Statistical analysis

No formal statistical hypotheses were set. Only descriptive analyses were performed. Continuous variables were reported as mean (SD) and median (25th–75th percentile), while categorical variables were reported as number and percentage of cases and distribution of frequencies.

PRO questionnaires scores were calculated according to the instructions obtained from copyright holders. The three TSQM‐9 domain scores (effectiveness, convenience, and global satisfaction), ranging from 0 to 100 with higher scores representing higher satisfaction, were calculated as primary analysis.^15^, ^16^, ^19^ In addition, a sensitivity analysis was performed for the primary objective providing treatment satisfaction among patients not previously treated with other antipsoriatic therapies (treatment‐naïve patients). Wilcoxon's test was used to compare TSQM‐9 domain scores between subgroups of patients having experienced previous antipsoriatic treatments at the time of treatment initiation with Cal/BD foam and treatment‐naïve patients. In order to describe predefined factors potentially associated to treatment satisfaction, the Spearman's correlation matrix and the Kruskal‐Wallis test were used for continuous variables (i.e., age at Cal/BD foam treatment initiation; duration of plaque psoriasis since first diagnosis) and categorical variables (i.e., gender; plaque psoriasis severity at enrolment), respectively. Regarding PPQ, higher single‐item scores reflect the presence of favorable adherence, influencing patient preference factors.^17^ TSQM‐9 and PPQ questionnaires used for LION study have been managed in agreement to the terms and conditions of each respective license obtained prior to start the data collection.

Statistical analyses were performed using SAS Enterprise Guide v. 7.1 and SAS 9.4 (SAS Institute, Cary, NC). Study design, electronic Case Report Form (eCRF) set‐up, and statistical analyses were performed by MediNeos S.U.R.L. (Modena, Italy), a company subject to the direction and coordination of IQVIA Solutions HQ Ltd, on behalf of LEO Pharma. Data management activities were performed in‐house by LEO Pharma according to their standard operating procedures.

## 3 RESULTS

### 3.1 Patients

Of the 258 patients enrolled in the LION study, 256 (99.2%) were considered fully eligible as per inclusion/exclusion criteria, whereas two patients were not considered eligible because they had signed the informed consent after closure of enrolment and were excluded from the analysis. One of the eligible patients was lost to follow‐up, but was evaluated for the time he remained in the study. In total, 255 patients completed the study. The median (25th–75th percentile) duration of observation was 30.0 (28.0–34.0) days. Patients had a mean (SD) age of 55.6 (15.4) years and were mostly male (n/N = 152/256, 59.4%). Mean (SD) duration of psoriasis since first diagnosis was 12.9 (14.0) years. Psoriasis severity was classified as mild in 133 (52.0%) patients, moderate in 111 (43.3%) patients, and severe in 12 (4.7%) patients according to medical judgment. Main clinical characteristics of the patients at enrolment, including psoriasis localizations, are reported in Table 1.

**TABLE 1:** Main clinical characteristics of eligible patients (N = 256) at baseline

	n (%)
Plaque psoriasis severity according to medical judgment	
Mild	133 (52.0)
Moderate	111 (43.3)
Severe	12 (4.7)
Signs and symptoms of psoriasis	
Erythema	135 (52.7)
Itching	128 (50.0)
Scaling of the skin	96 (37.5)
Skin cracking/dry skin	52 (20.3)
Burning	43 (16.8)
Joint pain	15 (5.9)
Skin pain	9 (3.5)
Nail pain	8 (3.1)
Rash	8 (3.1)
Bleeding	7 (2.7)
Fatigue	7 (2.7)
Swelling	1 (0.4)
Location of signs and symptoms of psoriasis	
Elbows	109 (42.6)
Trunk	98 (38.3)
Scalp	94 (36.7)
Legs (excluding feet and knees)	84 (32.8)
Knees	72 (28.1)
Arms (excluding hands and elbows)	63 (24.6)
Gluteus	44 (17.2)
Hands	37 (14.5)
Palms	21 (8.2)
Foot/feet	17 (6.6)
Face	15 (5.9)
Soles	15 (5.9)
Nails	14 (5.5)
Genital area	14 (5.5)
Presence of comorbidities	170 (66.4)
Hypertension	83 (32.4)
Hypercholesterolemia/Dyslipidemia	49 (19.1)
Diabetes	31 (12.1)
Cardiomyopathy	23 (9.0)
Psoriatic arthritis	20 (7.8)
Obesity	17 (6.6)
Autoimmune diseases	12 (4.7)
Anxiety	10 (3.9)
Hepatitis	6 (2.3)
Depression	5 (2.0)
Asthma	4 (1.6)
Other, specify	70 (27.3)

### 3.2 Treatments

Previous antipsoriatic treatments had been received by 80.5% of patients (n/N = 206/256), as detailed in Table 2. As a reason for the choice of Cal/BD cutaneous foam, the treating dermatologists reported primarily the clinical need to obtain a rapid response (n = 145, 56.6%), followed by the failure to the previous topical (n = 78, 30.5%) and non‐topical (n = 31, 12.1%) therapy, and lack of adherence to previous treatments (n = 26, 10.2%). Other reasons for treatment choice were the patients' clinical characteristics (e.g., presence of comorbidities, mild severity of psoriasis). The mean (SD) body surface area treated with Cal/BD foam was 9.7% (7.1). The treatment was applied to body areas including scalp or scalp only in 37.9% of patients (n/N = 97/256), whereas the remaining patients applied it to body areas excluding scalp. Twenty‐four patients (9.4%) received at least one concomitant antipsoriatic therapy, in particular phototherapy (n/N = 17/24, 70.8%) and topical corticosteroids (n/N = 5/24, 20.8%).

**TABLE 2:** Antipsoriatic treatments before calcipotriol/betamethasone dipropionate (Cal/BD) foam treatment initiation (eligible patients, N = 256)

	n (%)
Patients having received previous antipsoriatic treatments	206 (80.5)
Types of treatments	
Topical therapy	184 (71.9)
Phototherapy	58 (22.7)
Conventional systemic therapy	54 (21.1)
Biological therapy	12 (4.7)
Number of previous treatments	
1	187 (73.0)
2	9 (3.5)
3	7 (2.7)
4	2 (0.8)
5	1(0.4)

Cal/BD foam treatment mean (SD) duration was 30.6 (5.8) days and in 79.7% of patients (n/N = 204/256) the treatment was still ongoing at the end of the 4‐week observation period. The main reasons for treatment permanent discontinuation were end of planned treatment (n = 36; 14.1%) and target response reached (n = 22; 8.6%). Self‐reported adherence to treatment was full in 86.7% (n/N = 222/256) of patients; another 10.5% (n = 27) of patients covered ≥80% of prescribed days.

### 3.3 Primary objective: patients' treatment satisfaction

**FIGURE 1:**
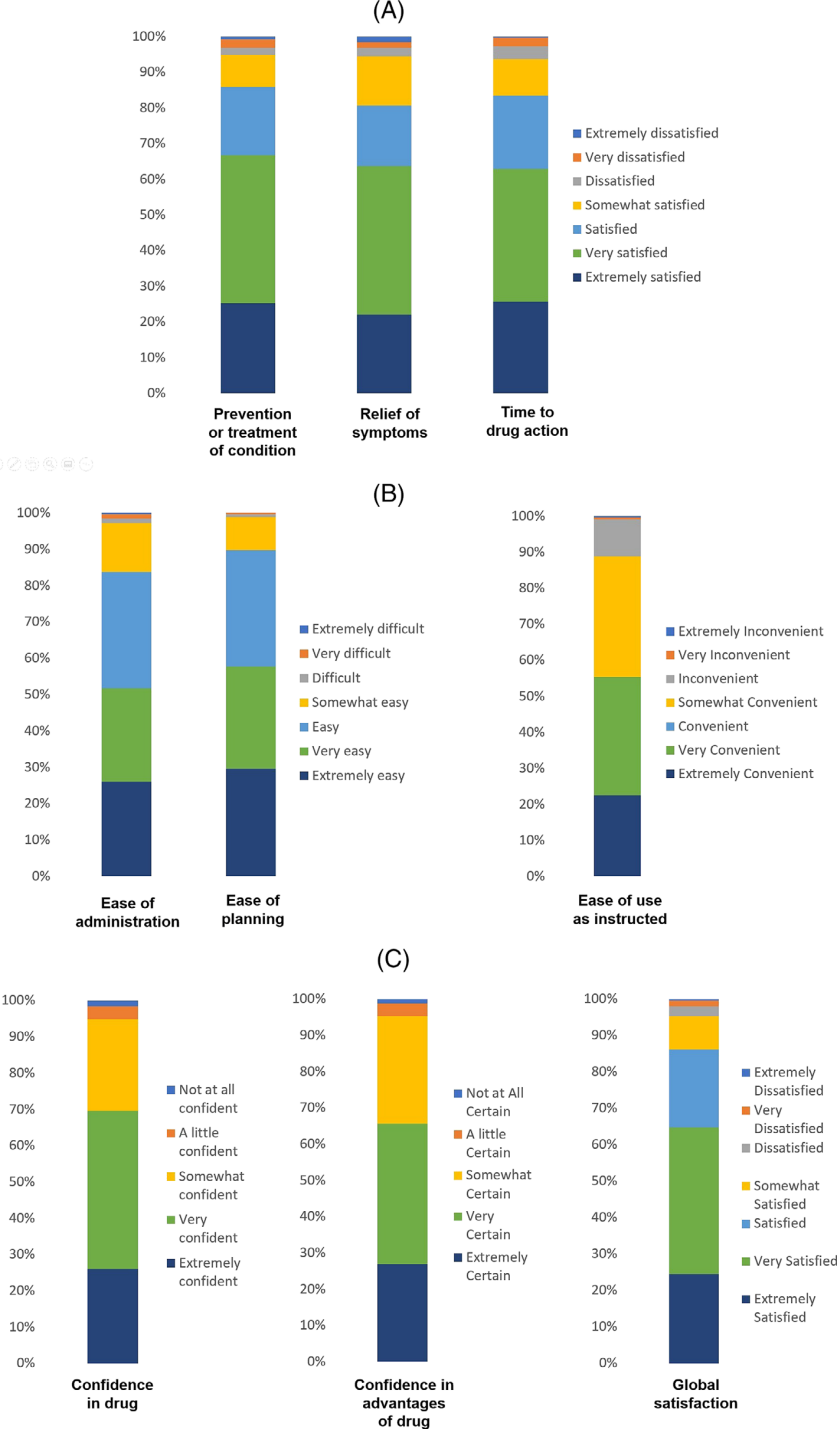
Patients' satisfaction after 4 weeks of calcipotriol/betamethasone dipropionate (Cal/BD) cutaneous foam treatment as measured by 9‐item Treatment Satisfaction Questionnaire for Medication (TSQM‐9): answers to the TSQM‐9 items, grouped by effectiveness domain (A), convenience domain (B), and global satisfaction domain (C)

A total of 253 patients overall out of the eligible 256 had evaluable data for TSQM‐9. High levels of patient's satisfaction with Cal/BD cutaneous foam measured by the TSQM‐9 were observed, with a median (25th‐75th percentile) satisfaction score of 83.3 (66.7–88.9) in the effectiveness domain, 77.8 (66.7–88.9) in the convenience domain and 78.6 (64.3–92.9) in the global satisfaction domain. In particular, 85.8% of patients were satisfied to extremely satisfied with the ability of the foam to prevent or treat their condition and 80.6% with the foam‐induced relief of symptoms; more than 80% of patients rated from easy to extremely easy to use and to plan when to use the Cal/BD foam (Figure 1). In addition, patients treated with Cal/BD foam on scalp with available TSQM‐9 data (n = 96) were satisfied with treatment similarly to the overall assessed patients.

Descriptive statistics for all three TSQM‐9 domains obtained at the 4‐week final visit in overall patients and in patients treated on scalps with available data are shown in Table 3.

**TABLE 3:** Patients' treatment satisfaction according to the 9‐item Treatment Satisfaction Questionnaire for Medication (TSQM‐9) domains (primary objective)

Domain		Effectiveness	Convenience	Global satisfaction
Score		(0–100)	(0–100)	(0–100)
Overall patients with available TSQM‐9 data	N	253	253	253
	Mean (SD)	77.1 (19.3)	77.7 (15.5)	74.6 (19.2)
	Median (25th–75th percentile)	83.3 (66.7–88.9)	77.8 (66.7–88.9)	78.6 (64.3–92.9)
Patients treated on scalp with available TSQM‐9 data	N	96	96	96
	Mean (SD)	75.2 (21.6)	77.6 (17.3)	73.6 (22.0)
	Median (25th–75th percentile)	83.3 (66.7–88.9)	77.8 (66.7–88.9)	78.6 (57.1–92.9)

Abbreviation: TSQM‐9, 9‐item Treatment Satisfaction Questionnaire for Medication.

### 3.4 Secondary objectives

**FIGURE 2:**
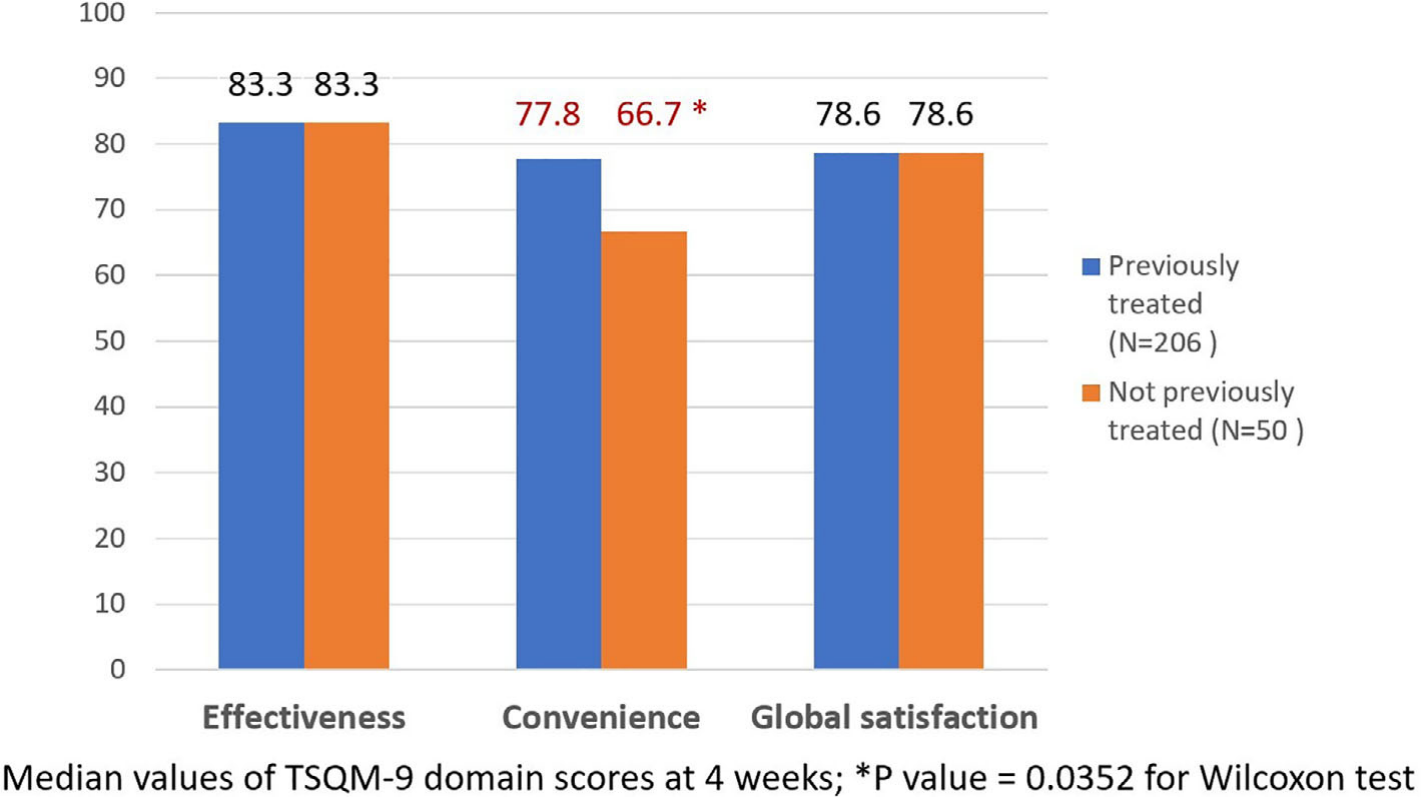
Patients' satisfaction after 4 weeks of calcipotriol/betamethasone dipropionate (Cal/BD) cutaneous foam treatment as measured by 9‐item Treatment Satisfaction Questionnaire for Medication (TSQM‐9): TSQM‐9 domains, stratified by previous treatment

As reported in Figure 2, median score for TSQM‐9 convenience domain was significantly (*p* = 0.0352 for Wilcoxon test) higher for evaluable patients previously treated with antipsoriatic treatments (n = 204) (median [25th–75th percentile] of 77.8 [66.7–88.9]) than for evaluable treatment‐naïve patients (n = 49) (median [25th–75th percentile] of 66.7 [61.1–88.9]). The median scores for global satisfaction and effectiveness TSQM‐9 domains were not significantly different.

In addition, the patient satisfaction regarding Cal/BD foam was not influenced by different gender, age, plaque psoriasis severity, and duration of the disease since first diagnosis. These considerations did not provide the assumptions to elaborate a multivariate statistical model including the predefined variables.

**FIGURE 3:**
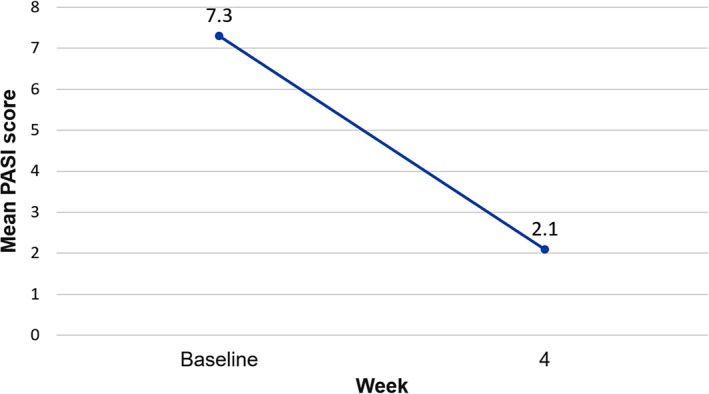
Intra‐patient change in mean Psoriasis Area Severity Index (PASI) score after 4 weeks of calcipotriol/betamethasone dipropionate (Cal/BD) cutaneous foam treatment with respect to baseline

**FIGURE 4:**
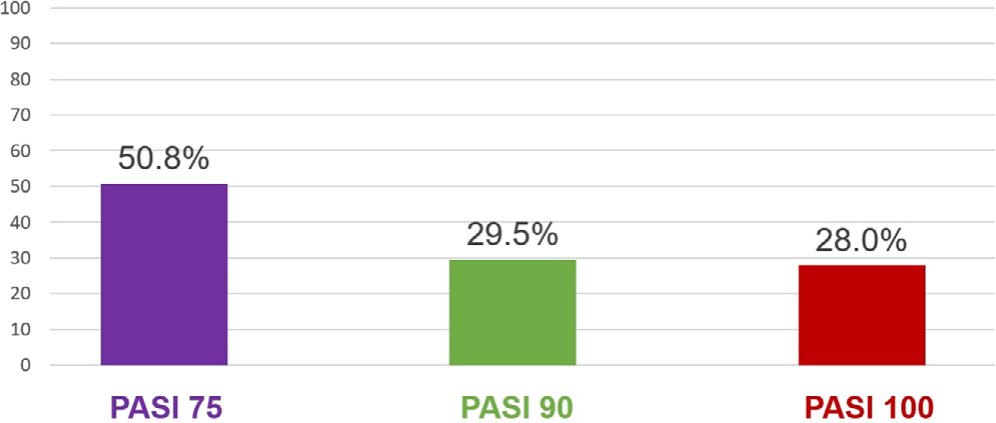
Psoriasis Area Severity Index (PASI) 75/90/100 response achievement (%) after 4 weeks of calcipotriol/betamethasone dipropionate (Cal/BD) cutaneous foam treatment among patients with PASI scores available both at baseline and at end of observation (N = 132)

Mean (SD) PASI score at Cal/BD foam treatment initiation was 7.3 (4.8); after 4 weeks, at study final visit, the mean value was 2.1 (2.7), with an intra‐patient change of −4.7 (3.0) as illustrated in Figure 3. PASI 75/90/100 response rates after 4 weeks of treatment are shown in Figure 4.

In patients who had been treated for psoriasis prior to the Cal/BD foam, the reasons for preferring the Cal/BD foam over previous therapies were assessed by PPQ. Regardless of the treatment previously received, most patients ‘agreed’ or ‘strongly agreed’ that Cal/BD foam was more effective, easier to use and associated with fewer side effects and better tolerability than previous treatments (Table 4 **)**.

**TABLE 4:** Patients' preference for the calcipotriol/betamethasone dipropionate (Cal/BD) foam treatment over the previous treatment by Patient Preference Questionnaire (PPQ) items (patients previously treated with antipsoriatic therapies, with applicable and available questionnaire data)

PPQ item	Patients in agreement/strong agreement n/N (%)
Cal/BD foam treatment versus previous topical treatment	
1–More effective	182/190 (95.8)
2–Easier to use	176/190 (92.6)
3–Fewer side effects	160/171 (93.6)
4–Better tolerable	176/186 (94.6)
5–Preferred	185/190 (97.4)
Cal/BD foam treatment versus previous systemic treatment	
6–More effective	79/110 (71.8)
7–Easier to use	94/109 (86.2)
8–Fewer side effects	96/105 (91.4)
9–Better tolerable	97/108 (89.8)
10–Preferred	96/109 (88.1)

Abbreviations: Cal/BD, calcipotriol/betamethasone dipropionate; PPQ, Patient Preference Questionnaire.

## 4 DISCUSSION

First‐line management of mild or moderate plaque psoriasis consists of topical treatments^20^; however, they are associated with lower adherence rates than systemic treatments, which represents a major issue for treatment effectiveness.^21^ Probably consistent with this, patients with plaque psoriasis are generally not very satisfied with their treatment.^8^, ^9^, ^22^ A successful topical treatment option should be able to achieve higher patient acceptability and adherence in order to improve signs and symptoms.

TSQM‐9 median values of global satisfaction and satisfaction for the effectiveness of Cal/BD foam after 4 weeks of therapy were extremely high and identical in both treatment‐naïve and previously treated patients, and did not differ according to patient's demographics or main psoriasis disease characteristics. Satisfaction for the convenience of the treatment was slightly lower but overall high, and was significantly higher among patients that had been previously treated for psoriasis. This suggests that experience with previous types of therapies increased the patients' satisfaction using Cal/BD foam regarding to convenience. The first study addressing patients' treatment satisfaction with the Cal/BD foam registered a proportion of overall satisfied patients (84%) comparable to that of our study.^23^ The ease of use of the Cal/BD foam had been previously reported in one Spanish study and a few other trials before.^12^, ^24^ It is well known that dissatisfaction with treatments results in poor adherence, which in turn impacts treatment effectiveness.^25^, ^26^ Conversely, in several different disease settings it has been reported that treatment satisfaction is associated with greater adherence, in particular, the global satisfaction and the convenience domains. ^27^, ^28^, ^29^, ^30^ Importantly, adherence to treatment in our study was remarkably high and the majority of patients remained on Cal/BD foam after study completion. The patients who discontinued treatment permanently did so by medical decision linked to the therapeutic strategy in place, and not due to adverse events.

The favorable results in terms of treatment satisfaction are consistent with those obtained at the PPQ, a tool that specifically assesses the patient's preference for the current treatment over previous ones. More than 97% of non‐naïve patients highly preferred Cal/BD foam over previously received topical treatments and between 92 and 96% of patients considered it more effective, better tolerated, and easier to use compared to previous topicals. Slightly less than 94% of patients also reported a perception of fewer side effects. These percentages are even higher than those obtained in the PSO‐ABLE clinical trial.^12^ However, it must be taken into account that these data are obtained in a patient population selected based on a need for treatment change (e.g., due to insufficient response or side‐effects with previous treatment). Therefore, these results cannot be generalized or attributable exclusively to the Cal/BD foam treatment.

As an objective measure of change in disease state, by psoriasis signs and symptoms, we collected absolute PASI scores and PASI response rates, although only as a secondary objective. Unfortunately, the number of patients with available PASI assessment both at enrolment and at end of the observation was limited (N = 132). The reason for this lies is in the fact that the outbreak of the Covid‐19 pandemic made it difficult to perform a 4‐week follow‐up visit at the clinic. In the patients with enrolment and end‐of‐observation PASI assessments available (N = 132), mean PASI score decreased after 4 weeks of Cal/BD foam treatment, with 50.8% (n = 67) of patients achieving a PASI 75 response and only 9.4% having received concomitant antipsoriatic treatments, mainly phototherapy. These data suggest a positive impact of the Cal/BD foam treatment on the severity and extent of the skin lesions. This is particularly interesting in the light of the fact that nearly half of the patient population had moderate or severe psoriasis and some of them had lesions in difficult‐to‐treat areas. The benefits of Cal/BD cutaneous foam in plaque psoriasis have been previously demonstrated in large randomized controlled trials, in terms of PASI, physician's global assessment (PGA), itch relief, and improved overall clinical assessment scores.^10^, ^11^, ^12^, ^13^, ^31^ In particular, consistently with our results, studies performed with the Cal/BD foam show PASI 75 response rates ranging between 49 and 52.9%,^32^ although most of the previous studies used the modified PASI (mPASI) that excludes the head. Improvement of psoriasis signs and symptoms with Cal/BD foam was also strongly perceived by our patients, who expressed an extremely high level of satisfaction in the effectiveness domain of TSQM‐9.

With regards to scalp psoriasis, data on the effectiveness of the Cal/BD foam is still scarce. A recent limited case series showed encouraging results in patients with a long history of scalp psoriasis.^32^ The fairly high proportion of patients in our cohort with scalp psoriasis (36.7%) and the overall positive results achieved in our study seem to suggest that these patients also benefited from and were satisfied with the Cal/BD foam treatment.

Our study has the limitations of a real‐life observational design, including selection bias and information bias. Study sites were not randomly sampled from the whole pool of Italian clinics; however, participating sites were selected across a variety of geographic regions and institutions, and patients were consecutively enrolled in order to minimize selection bias. A key selection bias was the enrolment of a population of patients needing to change their previous treatments, so that patients well treated and satisfied with other therapies were not included. The non‐random assignment of subjects to the Cal/BD foam treatment could lead to confounding by indication due to the phenomenon of channeling: patients enrolled in the study may have been among those insufficiently treated or particularly dissatisfied with prior antipsoriatic therapies and therefore channelized to the study sites. It should be taken into account that the PASI change from baseline was assessed in a subgroup of patients with available data at the follow‐up visit. In order to minimize missing data, follow‐up information other than PASI assessment was collected either at the clinical site or over the phone, according to the evolving clinical practice due to COVID‐19 pandemic. In particular, the study protocol allowed patients to fill in the TSQM‐9 and the PPQ in an out‐of‐hospital setting. Finally, it is acknowledged that the inclusion of patients in a study may alter their perception of medical care, generally in a more positive sense. Despite the above listed limitations, this prospective study provides real‐life evidence of the effectiveness and optimal acceptability of the topical treatment with the Cal/BD cutaneous foam in the Italian dermatological clinical practice.

## 5 CONCLUSIONS

This study shows that a cohort of patients with plaque psoriasis in a real‐practice setting was highly satisfied with a Cal/BD cutaneous foam topical treatment, mainly appreciating its effectiveness and convenience, regardless of whether patients were treatment naïve or had already been treated for psoriasis. Those who had received previous treatments reported high preference for the Cal/BD foam over previous topical therapies. In terms of objective assessments, the PASI results seem to confirm in the Italian dermatological daily practice the Cal/BD foam positive impact observed in controlled clinical trials.

## AUTHOR CONTRIBUTIONS

All authors meet criteria for authorship as recommended by the International Committee of Medical Journal Editors (ICMJE) and were fully responsible for all aspects of manuscript development. All authors together with the study group have made substantial contribution to investigation and acquisition of data.

## Data Availability

The data that support the findings of this study are available on request from the corresponding author. The data are not publicly available due to privacy or ethical restrictions.
